# Deep learning for cross-region streamflow and flood forecasting at a global scale

**DOI:** 10.1016/j.xinn.2024.100617

**Published:** 2024-03-26

**Authors:** Binlan Zhang, Chaojun Ouyang, Peng Cui, Qingsong Xu, Dongpo Wang, Fei Zhang, Zhong Li, Linfeng Fan, Marco Lovati, Yanling Liu, Qianqian Zhang

**Affiliations:** 1 State Key Laboratory of Mountain Hazards and Engineering Resilience, Institute of Mountain Hazards and Environment, Chinese Academy of Sciences, Chengdu 610299, China; 2 State Key Laboratory of Geohazard Prevention and Geoenvironment Protection, Chengdu University of Technology, Chengdu 610059, China; 3 Data Science in Earth Observation, Technical University of Munich, 80333 Munich, Germany; 4 State Key Laboratory of Loess and Quaternary Geology, Institute of Earth Environment, Chinese Academy of Sciences, Xi’an 710061, China; 5 Department of Civil Engineering, McMaster University, Hamilton, ON L8S 4L8, Canada; 6 Chengdu University of Information Technology, Chengdu 610225, China

**Keywords:** deep learning, hydrology, encoder-decoder double-layer LSTM, ED-DLSTM, streamflow forecasting, hydrological regionalization, cross-region

## Abstract

Streamflow and flood forecasting remains one of the long-standing challenges in hydrology. Traditional physically based models are hampered by sparse parameters and complex calibration procedures particularly in ungauged catchments. We propose a novel hybrid deep learning model termed encoder-decoder double-layer long short-term memory (ED-DLSTM) to address streamflow forecasting at global scale for all (gauged and ungauged) catchments. Using historical datasets, ED-DLSTM yields a mean Nash-Sutcliffe efficiency coefficient (NSE) of 0.75 across more than 2,000 catchments from the United States, Canada, Central Europe, and the United Kingdom, highlighting improvements by the state-of-the-art machine learning over traditional hydrologic models. Moreover, ED-DLSTM is applied to 160 ungauged catchments in Chile and 76.9% of catchments obtain NSE >0 in the best situation. The interpretability of cross-region capacities of ED-DLSTM are established through the cell state induced by adding a spatial attribute encoding module, which can spontaneously form hydrological regionalization effects after performing spatial coding for different catchments. The study demonstrates the potential of deep leaning methods to overcome the ubiquitous lack of hydrologic information and deficiencies in physical model structure and parameterization.

## Public summary

• An end-to-end model called ED-DLSTM with superior flood forecasting in gauged and ungauged catchments was proposed.
• For the first time, multiple hydrological AI models were trained and provided comparative analyses at a global-scale.
• The time-series forecasting capacities were apparently improved by the encoding of spatial attributes and transferability ability was well explained.

## Graphical abstract

**Figure:**
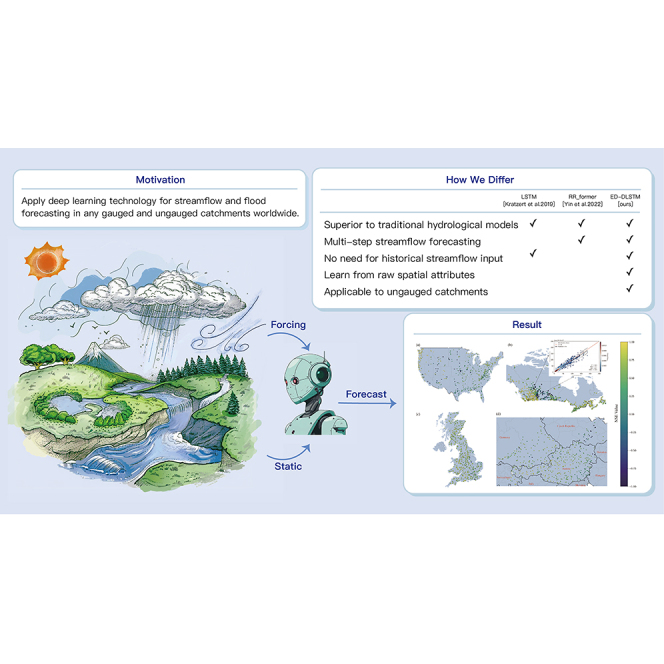

## Introduction

Artificial intelligence (AI) technology is rapidly transforming human lives at an unprecedented pace, including but not limited to the fields such as protein structure prediction in medicine,^1^^,^^2^ autonomous driving in transportation,^3^ and weather forecasting in meteorology.^4^ Recent progress and expansion in deep learning has led to a more sophisticated innovative technology, such as generative adversarial networks (GANs)^5^ for speech recognition, generative pretrained transformer (ChatGPT)^6^ for conversational assistance, and stable diffusion (SD)-based models like SORA or DALLE,^7^ for image and video generation. Meanwhile, AI for Science (AI4S) is profoundly changing every aspect of science as a new paradigm.^8^ For hydrological science, the AI technology is also providing an alternatively novel solution of streamflow and flood forecasting.

The primary methods for flood and streamflow forecasting can be classified into physical process-based methods (eg, soil and water assessment Tool (SWAT), système hydrologique européen (Mike SHE), and the hydrologiska fyrans vattenbalans modell (HBV model)^9^ and data-driven methods (eg, regression, fuzzy-based approaches, and artificial neural networks (ANN) methods).^10^ While physical models may be able to capture salient features of physical processes and underlying mechanisms controlling streamflow, their prediction skills and performance are critically dependent on parameter calibration. In contrast, data-driven models do not rely on prior catchment knowledge or assumptions regarding the system, instead, these methods learn to map directly from dynamic inputs and ancillary data to produce required outputs such as atmosphere and streamflow predictions.^11^^,^^12^ On the other hand, data-driven models are critically dependent on the quality of historical data highlighting the enormous challenge of developing reliable streamflow predictions for thousands of catchments without access to physical parameters or historical data. Nevertheless, developing national or regional flood forecasting strategies and efficient planning and operation of water resources must rely on prediction of streamflow from thousands of catchments that have neither physical parameterization nor historical record.

Among the established data-driven methods, long short-term memory (LSTM) is a popular framework for hydrologic modeling tasks, including but not limited to streamflow forecasting,^13^ stream temperature prediction,^14^ and dissolved oxygen prediction.^15^ However, taking streamflow forecasting as an example, most recent work^16^^,^^17^ has focused on how to construct local models to achieve precise prediction for a given region. For instance, Kratzert et al.^18^ used basic LSTM for streamflow prediction for the first time. Barzegar et al.^19^ proposed an improved CNN-LSTM model for water level prediction. Yin et al.^20^ implemented a Transformer-based streamflow prediction model called RR-Former, but its model relied on historical streamflow as input, making it impossible to achieve generalization in unmeasured catchments. According to bias-variance theory,^21^ predictions by these methods are specific to the region of calibration and offer limited applicability and transferability to other regions. What’s more, these studies were based on local regions; so far there is no universal evaluation for streamflow prediction on a global scale.

Considering the chronic lack of information and model applicability, we propose a novel cross-regional spatiotemporal ensemble model termed encoder-decoder-based double-layer long short-term memory (ED-DLSTM) model that fuses static spatial attributes and temporal forcing attributes to achieve cross-region streamflow forecasting (CSF). First, a grid of attributes provide input into a spatial 2D matrix, and salient features of a location and its adjacent locations can be obtained by residual convolution.^22^ Then, a spatial pyramid pooling (SPP)^23^ is utilized to map the matrix information of different regions to a fixed high dimension, which can spatially encode specific regions. Subsequently, an encoded vector is used as the initial cell state layer of the LSTM cells. To realize streamflow forecasting in different regions, an end-to-end network is trained by injecting forcing attributes of a specific time step. Finally, the network learns a mapping from dynamic time series to the observed streamflow under static attributes of the region. Thus, it yields coherent CSF capabilities that can be extended to continental scales and globally.

**Figure 1:**
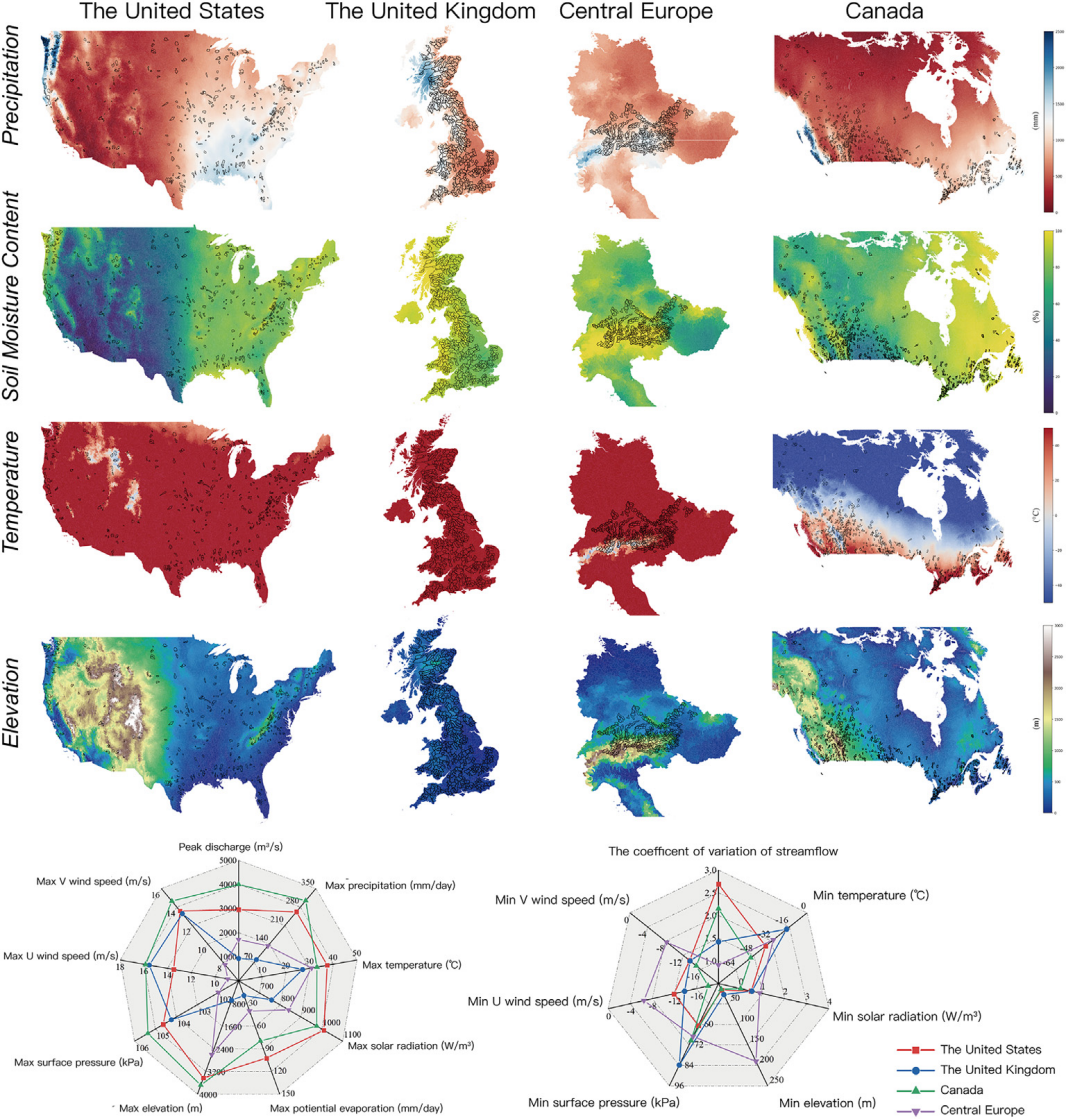
The distribution of catchment locations and records of several average annual variables from the period A.D. 1950–2000

Here, we selected four continental-level regions as study areas: the United States, Canada, Central Europe, and the United Kingdom, composing more than 2,000 catchments as illustrated in Figure 1. We trained four AI models to assess the performance of our CSF model (ED-DLSTM). Affected by Pacific airflow patterns, precipitation and soil moisture are high on the western coast of the United States and Canada. Overall, the eastern regions generally exhibit greater overall precipitation and soil moisture content compared with the west in the United States and Canada. The western and northern Scottish Highlands regions in the United Kingdom generally exhibit high average annual soil moisture content and precipitation, while the variability of other variables is comparatively low. In Central Europe, most of the Austrian region’s catchments have high relief, heavy precipitation, and low temperatures. The Rocky Mountains run through the United States and Canada. The basins near them have high terrain, high precipitation and soil moisture content, and low temperatures. Their complex evapotranspiration and snowmelt effects make the coefficient of variation of the streamflow even greater. We believe that the spatial variability in the above regions is large enough to verify the CSF capability of ED-DLSTM.

In the following we summarize the primary contributions of our numerical experiments.

(1) For the first time, multiple AI models were trained and provided comparative analyses for continental-scale streamflow forecasting using historical monitoring data. Compared with other models, ED-DLSTM demonstrates superior predictive capabilities.
(2) Capturing and encoding of spatial attributes make significant contributions to the skill of time series forecasting models in hydrology. Additionally, this process is easily performed and automated using machine learning technology.
(3) Even for significant differences in training data distribution, AI models can glean “general knowledge” for providing streamflow forecasts in ungauged catchments, an essential step for overcoming barriers to present streamflow prediction capabilities.

## Results

### A general model for streamflow forecasting in catchments with historical data

**Figure 2:**
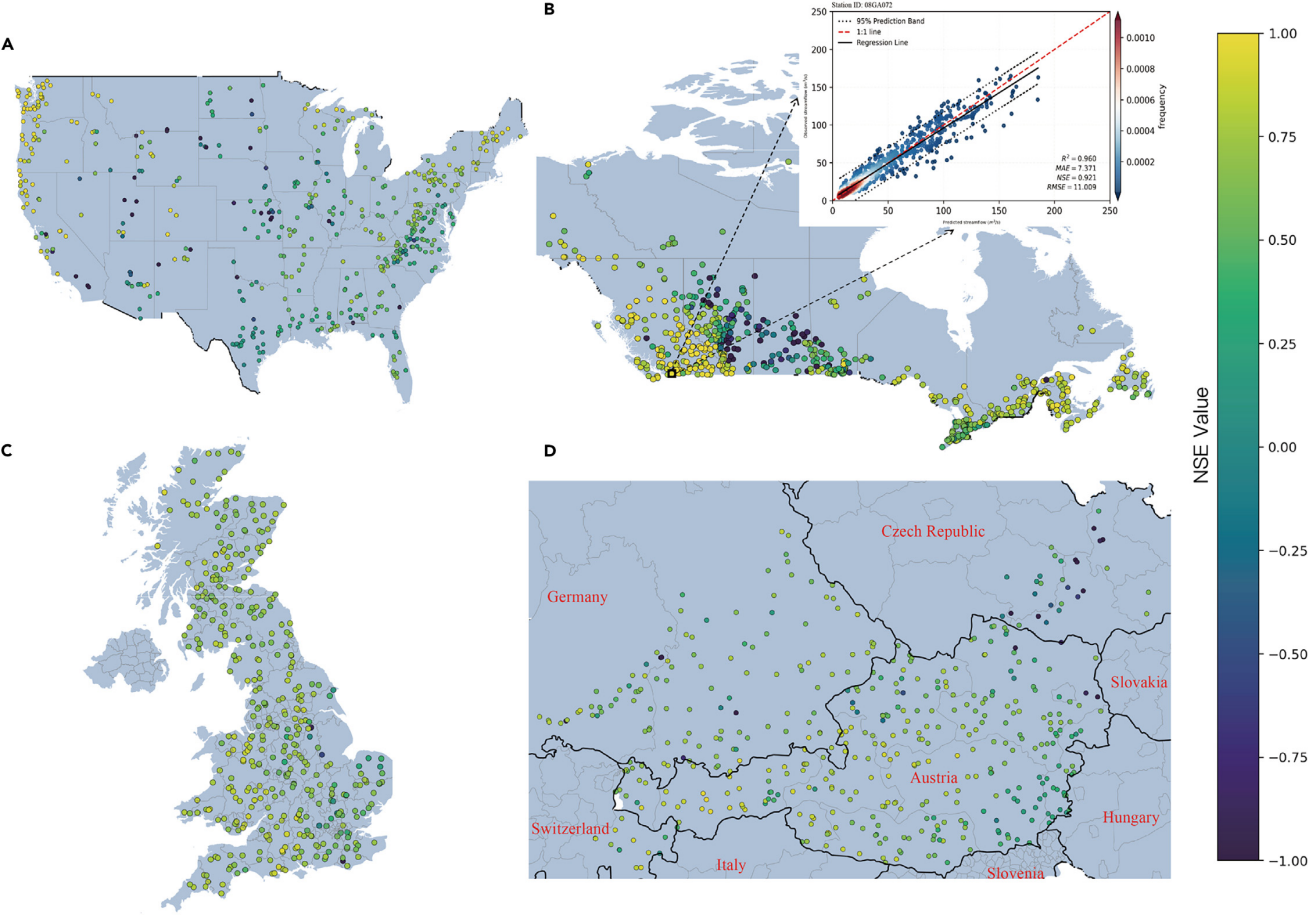
The NSE results produced by ED-DLSTM on the datasets (A) The United States. (B) Canada. (C) The United Kingdom. (D) Central Europe.

First, the prediction credibility of the ED-DLSTM model from January 1, 2010, to January 1, 2012, was evaluated on a comparative basis. In the United States, of the 482 catchments considered, 438 catchments achieved a Nash-Sutcliffe efficiency (NSE)^24^ greater than 0, with a mean NSE of 0.78 and a median NSE of 0.80. Similarly, in Canada, among the 740 catchments analyzed, 695 catchments achieved an NSE greater than 0, with a mean NSE of 0.80 and a median NSE of 0.82. For the United Kingdom, there were 406 catchments, of which 391 catchments achieved an NSE greater than 0, with a mean NSE of 0.68 and a median NSE of 0.70. In Central Europe, of the 461 catchments examined, 433 catchments achieved an NSE greater than 0, with a mean NSE of 0.73 and a median NSE of 0.79, as shown in Figure 2 (for better visualization, all NSE values less than −1 are reset to −1). Overall, those basins with heavier rainfall or larger runoff coefficient generally yield better prediction results. Remarkably, 81.8% of these basins achieved an average NSE higher than 0.6, underscoring the excellent prediction capacity and generalization in time of the ED-DLSTM model.

**Figure 3:**
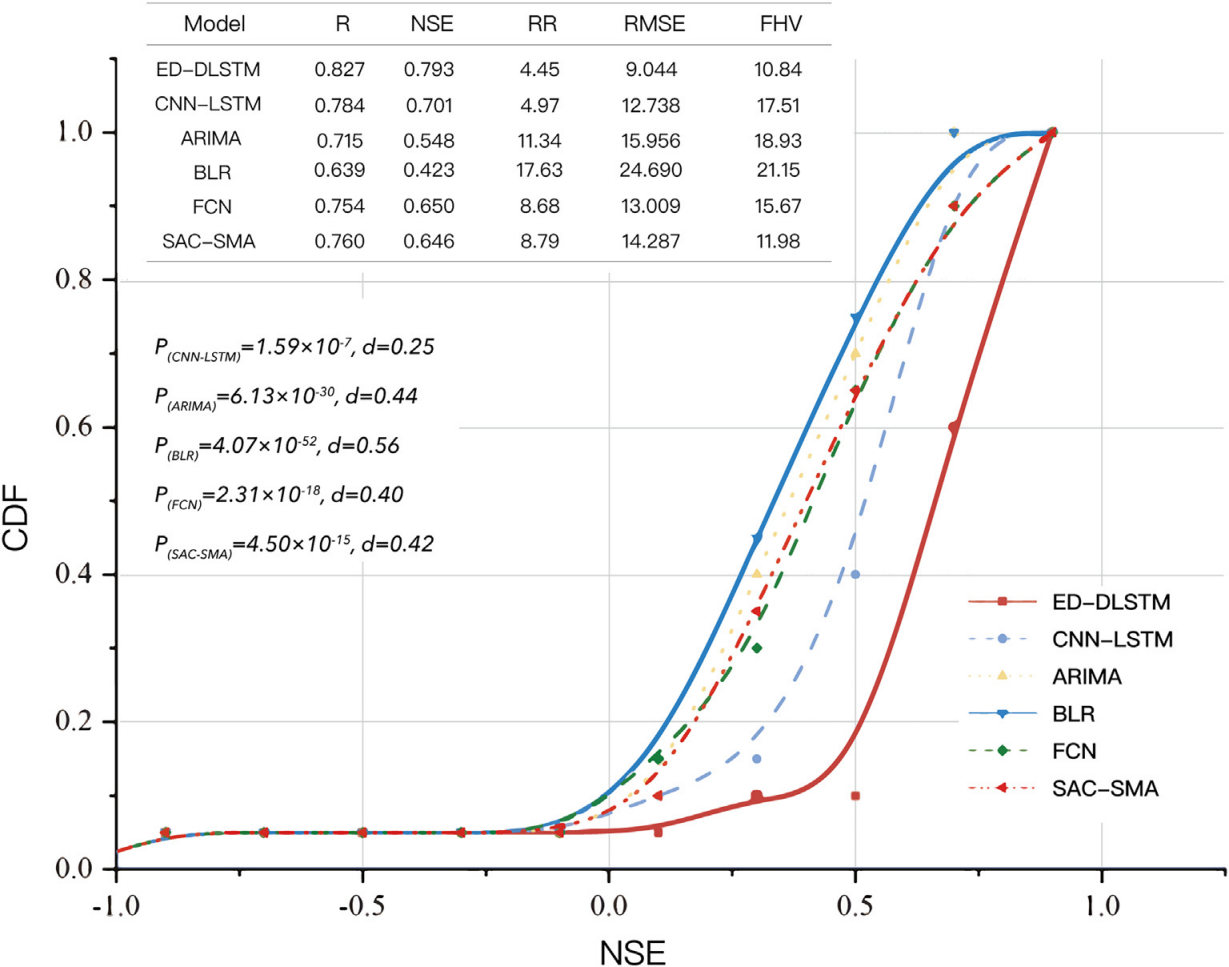
Cumulative density functions of the NSEs of ED-DLSTM, CNN-LSTM, ARIMA, BLR, FCN (data-driven methods) and SAC-SMA (process-based method)

At the same time, we compared the results of several other models mentioned in the section “Compared methods” in the United States region, as shown in Figure 3. As seen from the cumulative density function curves, our proposed model achieved state-of-the-art performance in CSF tasks relative to other machine learning models and classic hydrological models. We only used meteorological forcing and related static land variables to predict streamflow (note that the input does not include historical streamflow) in different regions. However, traditional data-driven models cannot distinguish between catchment attributes in different regions. In batches of deep learning samples, if the meteorological inputs of two catchments are similar but their streamflow values differ significantly, such a model will predict a mean value between the “label value” to minimize its loss, resulting in poor performance. Hydrologic models, such as Sacramento Soil Moisture Accounting (SAC-SMA), need to be physically calibrated for each catchment separately. This is an enormous challenge and a key obstacle in application of classical hydrologic models across large regions. The mean experimental results regarding the prediction performance achieved by different models in the test phase (from 2010 to 2012) are presented in the table in Figure 3. A Kolmogorov‒Smirnov significance test was performed on each model with ED-DLSTM. The *p* values were below the 0.01 significance threshold, supporting the rejection of the null hypotheses of identical distributions for the compared methods and ED-DLSTM. Among these models, CNN-LSTM ranked second, but it had a high peak flow deviation. The Fully Connected Neural Network (FCN) and SAC-SMA had similar levels; the average NSE of the FCN was higher, but its peak flow deviation was also higher. Autoregressive Integrated Moving Average Model (ARIMA) and Bayesian Multiple Linear Regression (BLR) performed well in a single catchment but ranked at the bottom in CSF tasks due to their lack of static inputs.

### Transferability of the model to ungauged catchments

In this section, we analyzed transferability of the proposed model to ungauged catchments for the period from January 1, 2010, to January 1, 2012. Most catchments have no historical observations, making forecasting of streamflow in such ungauged catchments a long-standing challenge. To address the challenge, we used the pretrained ED-DLSTM model (four regional models in the United States, Canada, the United Kingdom, Central Europe) to predict streamflow in catchments unstudied regions. Specifically, we selected 160 catchments in a region of central Chile ranging from the Atacama region in the North to the Lake district in the South to evaluate transferability of ED-DLSTM predictions. Data for the four pretrained regional models come from Northern Hemisphere countries, whereas the generalization test was conducted in Chile located in the Southern Hemisphere. It is believed the geographical and meteorological differences are significant^25^ enough to assess the generalization performance of the model on new data. ED-DLSTM did not train or fine-tune from any catchments located in Chile, and is evaluated solely using the observed streamflow data from these catchments. Therefore, the CSF ability assessment of ED-DLSTM in this region can be considered as transferability ability in ungauged catchments.

**Figure 4:**
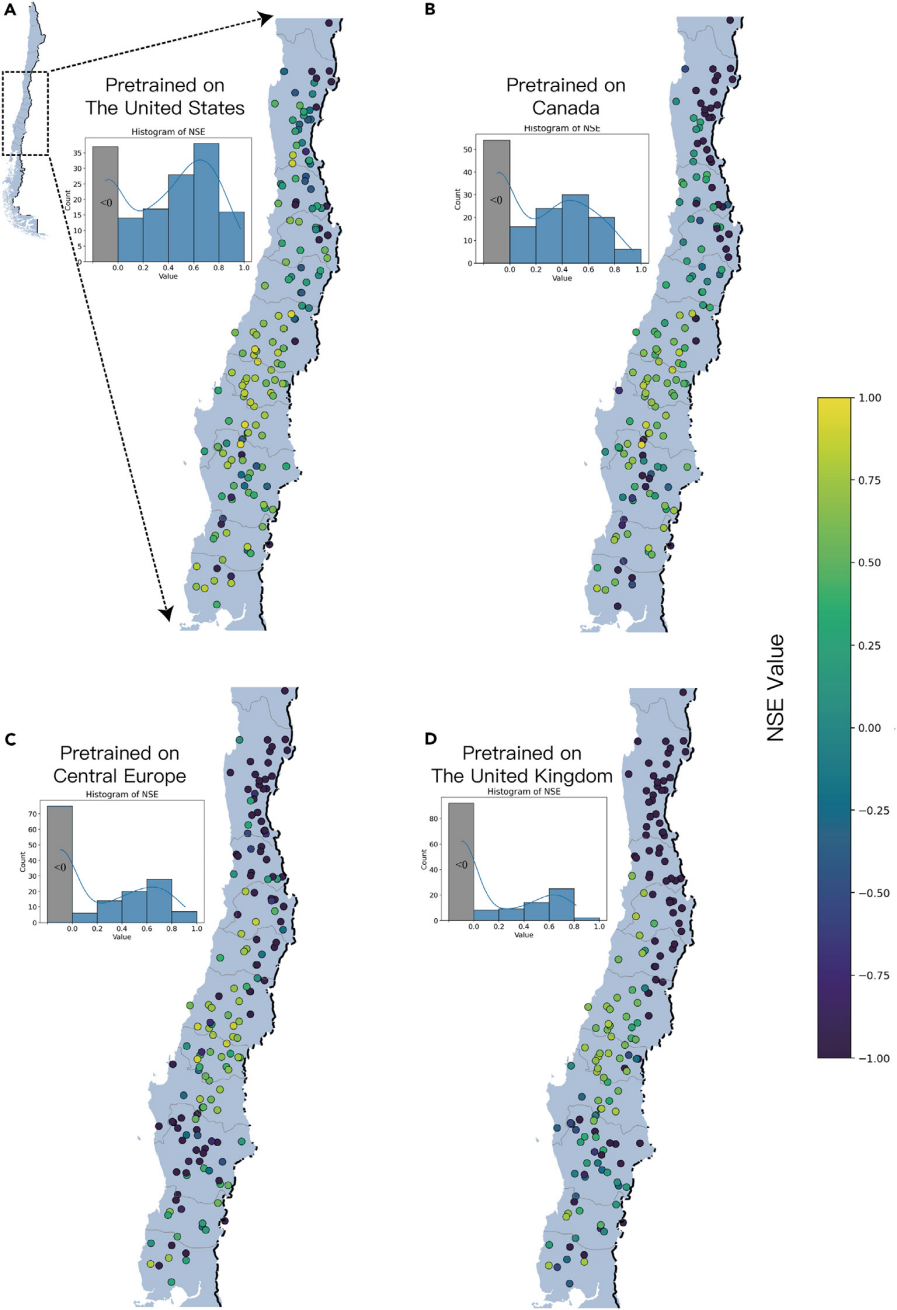
Model generalization results obtained in 160 new catchments in Chile (assumed to be ungauged) The gray columns included all catchments with NSE value less than 0.(A) Model pretrained on The United States. (B)Model pretraiend on Canada. (C) Model pretrained on Central Europe. (D) Model pretrained on The United Kingdom.

The prediction results for all catchments are presented in Figure 4, and histogram statistics were conducted. When deploying ED-DLSTM directly in the new region of Chile, the model pretrained on the United States demonstrates NSE greater than 0 for 76.9% of the catchments. The model pretrained on Canada achieves NSE greater than 0 for 66.2% of the catchments. The model pretrained on Central European achieves NSE greater than 0 for 53.1% of the catchments, and the model pretrained on the United Kingdom performs the poorest, with only 42.5% of the catchments exhibiting NSE greater than 0. (For better visualization, all NSE values less than −1 are reset to −1.) We speculate that the reason for the poorer performance of the model pretrained in the United Kingdom is because its data are more homogeneous and have smaller distribution differences, as shown in the radar plot in Figure 1. In this scenario, the simulation better fits the catchments within the training domain, but has poor generalization to fully new catchments, which is due to the variance-bias theory dilemma. In addition, ED-DLSTM exhibits good generalization in space. All models perform better in central and southern Chile (Maule, Biobío, and Araucaña regions) than in the north (Coquimbo Region), suggesting that the models seem to be able to learn a “hydrological general knowledge” from different catchments, resulting in consistent spatial distribution of model predictions in new catchments. As for the northern catchments, poor prediction results may be due to the impact of human activities that are not considered in the model or its distinct hydrological processes.

### Interpretability of the deep learning model

**Figure 5:**
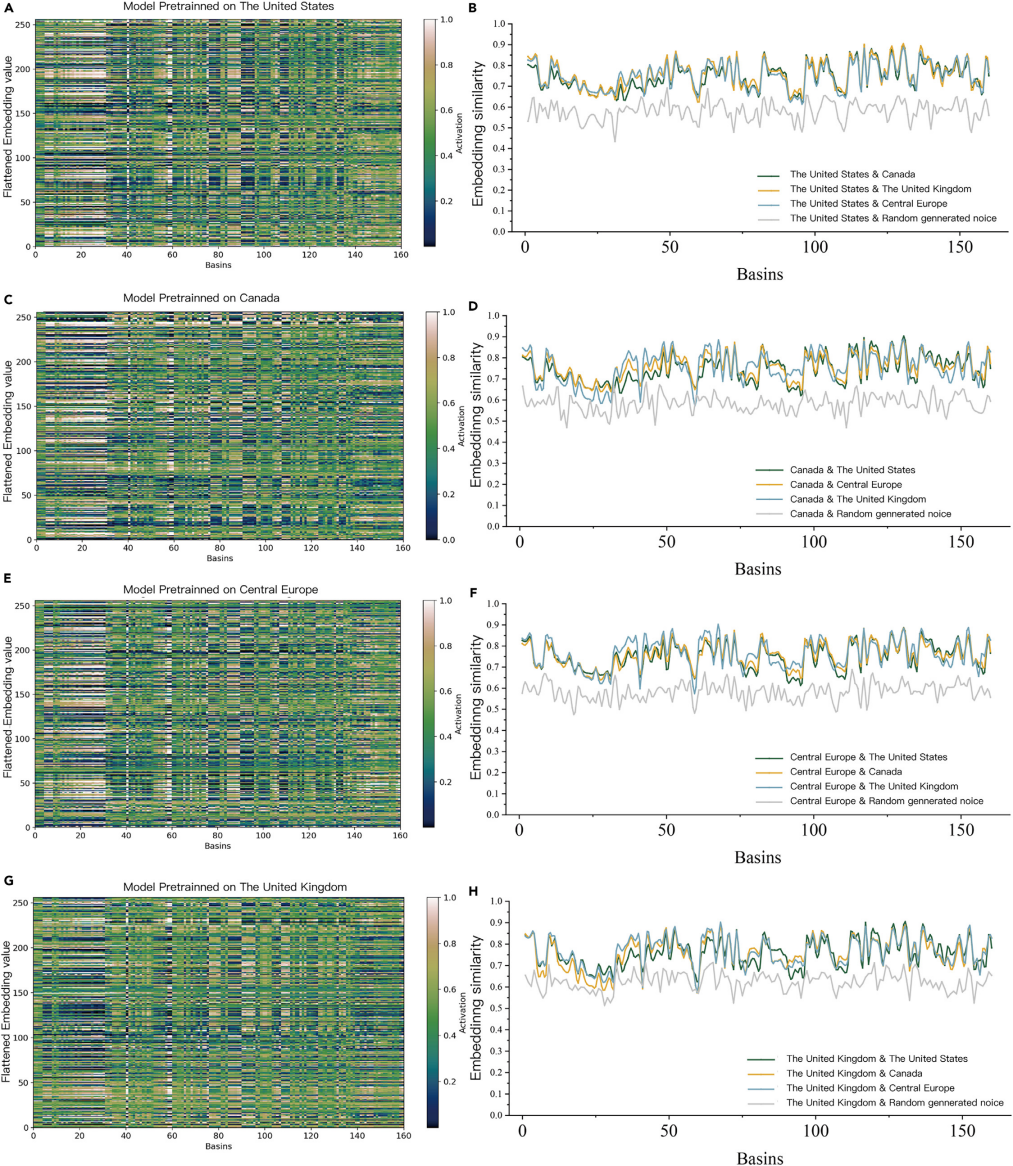
Parameters visualization and interpretability (A, B), (C, D), (E, F), and (G, H) indicate the model pretrained on The United States, Canada, Central Europe and The United Kingdom, respectively. Left part shows the visualization of flattened encoding features C_0_. Right part shows the tracking analysis of the embedding layer similarity between multiple models.

One of the biggest debates in deep learning is its interpretability. In many cases, the correspondences between inputs and outputs are black-box processes. Inspired by Jiang et al.,^26^ we tried to explain the hydrological behavior of the proposed model through the changes exhibited by its internal network parameters. As mentioned in the section “Encoder,” the initial cell state values C_0_ were derived from the upstream frameworks SPP, and each catchment was represented by a high-dimensional and unique vector (R ∈ 256), which reflected the model’s cognition of the catchments. This enabled us to intuitively see how the model “recognizes” different catchments and identifies them. As shown in Figures 5A, 5C, 5E, and 5G, the internal parameters of the C_0_ value of 4 pretrained model when deployed in the Chilean catchments are flattened and visualized, which intuitively show how the model uses information about catchment static characteristics to differentiate between various hydrological behaviors. It can be found that the encoded information of different regions changes significantly, which indicates that the model is able to distinguish the hydrological behaviors of different catchments. When the different pretrained models are deployed in the same catchment, the coding shows similarities, suggesting that deep learning techniques can regionalize the catchments. To verify this viewpoint, we conducted similarity analysis mentioned in the section “Model input selection and interpretability” on the encoding features of each region model in sequence, as shown in Figures 5B, 5D, 5F, and 5H, to verify whether the model can learn a certain degree of hydrological knowledge in different regions instead of overfitting the data within the training domain. It can be seen that the embedding layer similarity of each pretrained model shows a high degree of correlation, which verifies the view that the model can learn universal hydrological behaviors on different training sets. At the same time, we added a comparison of noise signals generated from Gaussian distribution to each model, and it was found that the similarities between pretrained models were always much greater than those of noise signals, these phenomena strengthen our trust in data-driven methods to a certain extent. According to statistics, the average embedding similarity between pretrained models is 38.4% greater than that between random generated noise, which proves from another perspective that the coding information of the catchments is not a random signal in disorder, but rather high-dimensional feature information recognized and utilized by the model.

## Discussion

In recent years, numerous studies have demonstrated the difficulty of surpassing the performance of deep learning models based on LSTM in hydrological simulation.^27^ However, most of them are based on local regions, and the uncertainty of the data limits the application on a larger scale. We proposed an ED-DLSTM model to “recognize” different catchments when encoding their regional spatial attributes, thus achieving high-precision streamflow forecasting. The model inputs include spatial static attributes and meteorological forcings that can well distinguish different hydrological behaviors. Our model is an end-to-end deep learning-based CSF model that outperforms the single-sequence time series model (LSTM) and several other benchmark models. Furthermore, the ED-DLSTM model only needs static attribute inputs to generate feature vectors, and these spatial attributes can be obtained from satellite data that are available worldwide.

Generally, researchers can manually build hierarchical structures for different catchments via clustering.^28^ We find that ED-DLSTM with spatial information coding will spontaneously make a similar choice; that is, the model can encode differences and similarities between basins. One explanation is that the unique spatial module of ED-DLSTM forces it to regionalize catchments through clustering to minimize the incurred losses.^29^ For example, two catchments with contrasting hydrological property will produce different streamflow values after experiencing the same rainfall duration (similar rainfall patterns), but the previously developed methods could not distinguish the difference between these two cases in the database.

For the first time, we have compared and verified multiple continent-level streamflow prediction AI models at the same time. Despite significant differences in weather patterns and hydrological responses among the four regional models in this case, the inclusion of spatial attribute encoding allows the models to acquire “general hydrological knowledge” from the data. This general knowledge enables the models to be applied to new, ungauged catchments to a certain extent. However, the impact of C_0_ values on the whole process still remains unknown because the encoded features of each catchment are represented by high-dimensional and nonlinear vectors that are adaptively generated by neural networks rather than explicit features. For example, similar catchments determined by the model may be the result of the joint influence of similar rainfall-runoff ratios, concentration times, soil retention capacities, hydrological balance patterns, etc. This is largely attributed to the accuracy-interpretability dilemma,^30^^,^^31^ which is worth further exploration in future work. Meanwhile, the prediction of hydrological response and flood risk under climate change is also highly worthy to be concerned in the models.^32^^,^^33^^,^^34^

## Materials and methods

### ED-DLSTM framework

**Figure 6:**
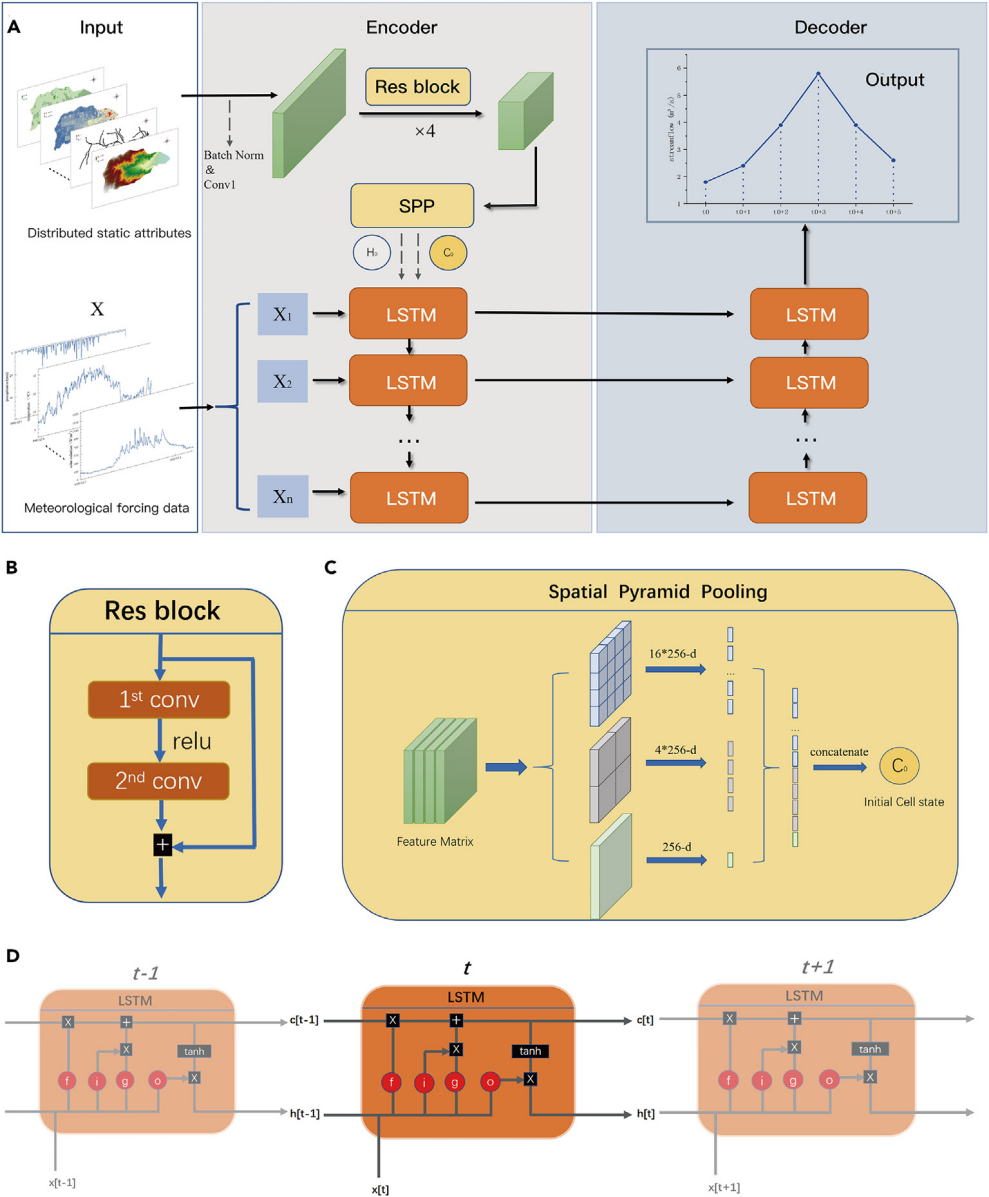
The framework of the proposed ED-DLSTM model (A) The overall structure of ED-DLSTM. (B) Schematic diagram of the Res-Block submodule. (C) The structure of the SPP layer. (D) The working principle of a single-layer LSTM model.

Figure 6A shows the overall structure of our proposed ED-DLSTM model for CSF. The encoder-decoder structure of our proposed ED-DLSTM model, which consists of two sub-models that operate in a symbiotic fashion,^35^ is more suitable for capturing global and local relationships through joint modeling. Our input data were multimodal, and the input spatial static grid attribute data formed a relatively sparse matrix. To extract physical properties, we adopted a multichannel 2D residual convolutional neural network in this case. In addition, the spatial grid resolutions of regions with different scales are different in actual situations. Therefore, we utilized an SPP module to obtain a convolutional feature matrix and map the multiscale spatial static properties of each region to a fixed vector. For example, when we set R ∈ 256, a single catchment was encoded as a vector with a length of 256 dimensions.

### Data collection

There are two types of input data: static and forcing data, as shown in Table 1. Static is geophysical data (non-time series) that provides information such as soil characteristics, or elevation. On the other hand, forcing data are dynamic time series data, such as precipitation, or temperature. Forcing name stems from the fact that these data are needed to run, or force, the model. Although static variables are not truly static, for practical purposes, they can be considered as relatively unchanging over shorter time periods, especially when compared with forcing information. Therefore, it is generally acceptable to treat static data as unchanged.

**Table 1:** Static and forcing attribute inputs that were used in our model

Types	Variable name	Description	Units
Static	Elevation	Digital elevation model with a 3 arc-second spatial resolution	m
	Snow Cover Extent	Snow cover extent with a 15 arc-second spatial resolution	%
	Soil Water Content	Soil layer thickness with a 30 arc-second spatial resolution	%
	Groundwater Table Depth	Groundwater table depth with a 30 arc-second spatial resolution	m
	Potential Evapotranspiration	The potential amount of evapotranspiration with a 30 arc-second spatial resolution	mm
	Channel Shape	Channel geometry morphology with a 3 arc-second spatial resolution	–
	Aridity Index	The degree of dryness and wetness with a 30 arc-second spatial resolution	–
Forcing	Precipitation	Daily total precipitation	mm
	Solar Radiation	Daily total solar radiation	W/m^2^
	Temperature	Daily min/max and mean temperature	°C
	Dew Point	Daily dew point temperature	°C
	Surface Pressure	Daily mean Surface pressure	kPa
	U Wind Component	Daily mean wind speed along the East direction	m/s
	V Wind Component	Daily mean wind speed along the North direction	m/s

The static attributes were derived from HydroATLAS^36^ and included digital elevation models (DEMs), snow cover extent, soil water content, groundwater table depth, potential evapotranspiration, aridity index, and channel geometry, which guided the model to distinguish the hydrological behaviors of different regions. The channel shape could be considered the river morphology, which was obtained by a spatial analysis based on the DEM data.

The forcing included precipitation, solar radiation, air temperature, dew point temperature, surface pressure, eastern and northern wind speed with a temporal resolution of 24 h and derived from ERA5-Land reanalysis.^37^ Normalization^38^ was performed to allow the network to efficiently learn nonlinear mapping relationship. These indicators were finally selected as inputs after considering accessibility and independence.

The model output is the prediction of streamflow at multiple time steps. Streamflow observations in all catchments are based on monitoring hydrological stations and merged into Caravan datasets,^39^ including CAMELS in the United States,^40^ HYSETS in Canada,^41^ LamaH-CE in Central Europe,^42^ and CAMELS-GB in the United Kingdom.^43^

### Encoder

Our encoder reasonably combines static and forcing information. First, the static part adopts ordinary convolution to integrate channels and then uses residual convolution to extract spatial static properties. Next, the spatial information of the high-level features is fed into the spatial pooling layer to obtain a region encoding that matches the dynamic LSTM input.

For any seven-channel input with an m∗n resolution, a standard convolution with a size of 3∗3@64 is used to integrate the channels and then batch-norm and input them into Res-Blocks, as shown in Figure 6B. x represents the residual mapping to be learned, which consists of two convolution layers $W_{i}$, each with an adjustable stride plus a ReLU rectification layer, as shown in Equation 1. In view of the sparseness of the spatial static attribute matrix, residual convolution can effectively avoid the gradient disappearance problem on the basis of preserving the relationship between the neighborhood and the local characteristics of the space. We adjust the down-sampling ratio of each block to 1/2, and the number of channels is successively doubled. After four consecutive Res-Blocks, a feature matrix of size (m/16)∗(n/16)∗256 is obtained. This feature matrix is then fed into the SPP layer to fix the encoding dimension.

(Equation 1) $$y=F_{c}\left(x,\left\{W_{i}\right\}\right)+x$$

Figure 6C shows the principle of integrating the encoded information into the SPP layer. The input of this module is the feature matrix extracted by the Res-Blocks. This module provides multilevel containers to respond to global or local biased feature extraction results. For example, for any feature matrix of size m'∗n'∗c, each level of the feature pyramid pooling window size is set as win=[m‘/α], the window sliding step str=[n‘/α] (α is the sub-grid scale, and the α structure adopted in this paper is 4, 2, 1), and the pooling (maximum/average) coding results of three scales can be obtained. Then, the features of each level are dimensionally spliced so that a feature matrix with any input size can be mapped into a 5,376 (16∗256 + 4∗256 + 256)-dimensional (high-dimensional) space. Finally, a multilayer perceptron is selectively used to convert the dimensions of this space to a low-dimensional spatial representation. This representation is used as the initial cell state *C*_*0*_ of the double bidirectional LSTM module to encode the spatial static properties of a specific catchment.

### Double and bidirectional long short-term memory layer

To perform CSF tasks, we optimize the basic LSTM model and design a double bidirectional LSTM model to better capture and model periodic time series terms, seasonal terms, cyclic terms, and irregular fluctuation terms, where the tensor flow within a layer is shown in Figure 6D. Specifically, a sequence $X=\left[x_{1},\ldots ,x_{n}\right]$ with any length n (n is 270), where x ∈R_m_ (m is 9, and it includes precipitation, solar radiation, minimum, maximum and mean temperature, dew point, surface pressure, eastern and northern wind speed), is input into each cycle unit in internal sequence order. At any time step t (1≤t≤n), the detailed internal processing mechanism of a dynamic forcing $x_{t}$ is as follows.

First, we calculate the input gate $i_{t}^{+}$, forget gate $f_{t}^{+}$, candidate hidden state $g_{t}^{+}$, and output gate $o_{t}^{+}$ within the forward LSTM cell, as shown in Equations 2, 3, 4, and 5, respectively.

(Equation 2) $$i_{t}^{+}=\sigma \left(W_{i}^{+}\left[h_{t-1}^{+},x_{t}\right]+b_{i}^{+}\right)$$

(Equation 3) $$f_{t}^{+}=\sigma \left(W_{f}^{+}\left[h_{t-1}^{+},x_{t}\right]+b_{f}^{+}\right)$$

(Equation 4) $$g_{t}^{+}=\tanh\left(W_{g}^{+}\left[h_{t-1}^{+},x_{t}\right]+b_{g}^{+}\right)$$

(Equation 5) $$o_{t}^{+}=\sigma \left(W_{o}^{+}\left[h_{t-1}^{+},x_{t}\right]+b_{o}^{+}\right)$$

Subsequently, we update and calculate the cell state $c_{t}^{+}$ and hidden state $h_{t}^{+}$ of the forward LSTM layer, as shown in Equations 6 and 7, respectively.

(Equation 6) $$c_{t}^{+}=f_{t}^{+}⊙c_{t-1}^{+}+i_{t}^{+}⊙g_{t}^{+}$$

(Equation 7) $$h_{t}^{+}=o_{t}^{+}⊙\tanh\left(c_{t}^{+}\right)$$

After that, we continue to calculate the input gate $i_{t}^{-}$, forget gate $f_{t}^{-}$, candidate hidden state $o_{t}^{-}$, and output gate $o_{t}^{-}$ of the reverse LSTM cell with the combination of forward information, as shown in Equations 8, 9, 10, and 11, respectively.

(Equation 8) $$i_{t}^{-}=\sigma \left(W_{i}^{-}\left[h_{t+1}^{-},x_{t}\right]+U_{i}^{-}h_{t}^{+}+b_{i}^{-}\right)$$

(Equation 9) $$f_{t}^{-}=\sigma \left(W_{f}^{-}\left[h_{t+1}^{-},x_{t}\right]+U_{f}^{-}h_{t}^{+}+b_{f}^{-}\right)$$

(Equation 10) $$g_{t}^{-}=\tanh\left(W_{g}^{-}\left[h_{t+1}^{-},x_{t}\right]+U_{g}^{-}h_{t}^{+}+b_{g}^{-}\right)$$

(Equation 11) $$o_{t}^{-}=\sigma \left(W_{o}^{-}\left[h_{t+1}^{-},x_{t}\right]+U_{o}^{-}h_{t}^{+}+b_{o}^{-}\right)$$

Finally, the cell state $c_{t}^{-}$ and hidden state $h_{t}^{-}$ of the inverse LSTM layer are calculated, as shown in Equations 12 and 13, respectively.

(Equation 12) $$c_{t}^{-}=f_{t}^{-}⊙c_{t+1}^{-}+i_{t}^{-}⊙g_{t}^{-}$$

(Equation 13) $$h_{t}^{-}=o_{t}^{-}⊙\tanh\left(c_{t}^{-}\right)$$

The above is the formula expression of the double bidirectional LSTM model, where + and − represent the forward and reverse directions of LSTM, respectively, σ represents the sigmoid function, ⊙ represents the elementwise multiplication operation, tanh represents the hyperbolic tangent function, and W, W and b represent learnable weight and bias parameters, respectively.

### Decoder

The decoder is responsible for mapping the high-level features to predicted streamflow values using an inverse LSTM layer. We choose to perform streamflow mapping in the last LSTM unit because the complete information of the Seq2Seq model should be decoded at the end, and this decoding layer can capture the information trend in reverse. We can separately encode and decode different hydrological response behaviors for various catchments. During the model training process, the network node values are iteratively updated using backpropagation,^44^ leading to convergence and the achievement of CSF.

### Compared methods

To validate our proposed model, we compared the performance of the proposed method with the following methods (four data-driven methods and one process-based method).

CNN-LSTM Model.^19^ This is a more powerful time series prediction model than the basic LSTM model. A 1-D convolution is used to extract the features of time series and then input them into the LSTM module to achieve streamflow forecasting.

ARIMA Model.^45^ The differential autoregressive moving average model is an early and widely used time series analysis model. Its performance is better when the input is a stationary time series.

Bayesian Multiple Linear Regression (BLR) Model.^46^ BLR regards the parameters of a linear model as random variables and uses the prior of the model to calculate its posterior; it is one of the most popular statistical methods.

Fully Connected Neural Network (FCN) model.^47^ The multidimensional input is mapped to the intermediate hidden layer, and then a high-dimensional hidden layer is mapped to the predicted value through an activation function.

Sacramento Soil Moisture Accounting (SAC-SMA) Model.^48^ This is a continuous soil moisture accounting model with spatially lumped parameters that simulates runoff within a catchment.

### Implementation details

We systematically divided the data into three parts: the training set, the testing set, and the validation set, as shown in Table S2. The training set of each region trains one model separately, aims to enable the model to learn hydrological relationships and latent features among different regions, while the testing set and validation set are used for model generalization testing. The generalizability performance of the ED-DLSTM includes generalization in time and generalization in space.^49^^,^^50^ Generalization in time refers to the model utilizing historical data from all catchments in a region for training and making predictions for future time periods in these catchments (time generalization is based on test set, as described in the section “A general model for streamflow forecasting in catchments with historical data”). Generalization in space refers to the model being trained on catchments within a specific region and making predictions for catchments in other new regions (space generalization is based on validation set, as described in the section “Transferability of the model to ungauged catchments”). To ensure the construction of standard sequence lengths, missing values in certain sites were removed, and sequences were replicated using the broadcast mechanism in Numpy. If there are only missing values throughout a time period that exceeds the length of the input sequence, the corresponding samples are excluded from the datasets. The experiments were implemented in 4 T V100 GPUs (32GB memory) with Pytorch (version 1.7.1). The batch-size was 64, and the number of epochs was 150. The Adam^51^ optimizer was used. To match the encoding results of the upstream framework, the dimensionality of the intracellular hidden layer of the LSTM model was set to 256. In addition, to avoid network overfitting, we adopted *L2* regularization and a dropout rate of 0.3 in the middle layer of the network. The initial learning rate was set to 0.001. In addition, a learning rate optimization scheme of cosine annealing^52^ was configured to help the network accelerate the subsequent convergence process.

(Equation 14) $$WLoss=\frac{1}{n}\sum_{i=1}^{n}w_{i}\left(\left|y_{i}-\hat{y_{i}}\right|\right)+\frac{1}{n}\sum_{i=1}^{n}\frac{w_{i}{\left(y_{i}-\hat{y_{i}}\right)}^{2}}{{\left(\sigma_{b}+\delta \right)}^{2}},i\in \left\{1,2,3,4,5\right\},\delta =1e-3$$

To guarantee the model learned stable trends in long-term series, we set the length of each meteorological input sequence to 180 days to predict the streamflow for the next 5 days (lead time can be set to 1–5 days). The above evaluations only analyzed the case in which the lead time is 1, and the analysis process at different lead times is the same. Furthermore, the mean and variance of flow in different catchments vary greatly. In order to avoid the model giving priority to high streamflow catchments to minimize errors, we utilized a weighted loss (WLoss) with two norm weights during training to dynamically improve the prediction results obtained at different lead time steps. The prediction of any basin will be divided by its streamflow variance $\sigma_{b}$ as a penalty during training, and a min value δ will be added to denominator to prevent zero division. In this way, the model will not rely on specific basins and focus on global optimization. The Nash efficiency coefficient^24^ (NSE) was used to evaluate the models. The proportions of daily prediction weights were different. Generally, the closer the lead time is, the larger the penalty coefficient should be, and the entropy increases with the passage of time. Less reliable prediction results yield lower penalty coefficients. In our experiments, the penalty coefficient $w_{i}$ was set to 0.3, 0.2, 0.15, 0.1, and 0.07.

### Model input selection and interpretability

**Figure 7:**
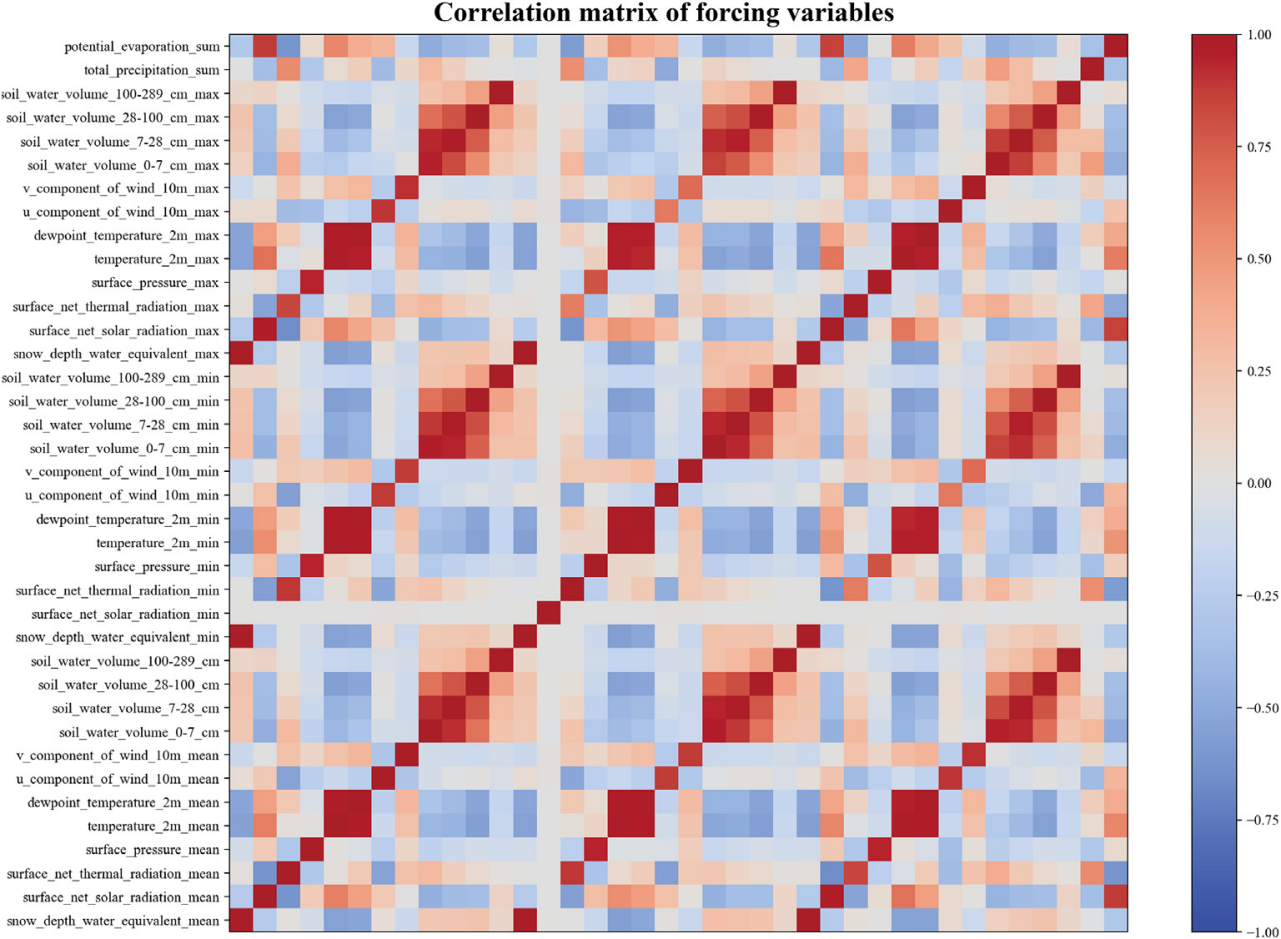
The correlation matrix of relevant forcing variables in ERA5-Land reanalysis products

In deep learning models, feature selection is crucial and directly affects the performance and complexity of the model. As shown in Figure 7, the correlations of ERA5-Land reanalysis variables with daily resolution (from 1981 to 2010) that can be used as model time series inputs are analyzed. The area-weighted spatial average for each variable was computed in each catchment area from spatial data (∼9 km spatial resolution) and shifted the time series (natively at GMT+0) to the local time of each gauge. In CSF task, the input variables should be as independent as possible and have low correlation. Taking into account their accessibility, the seven meteorological input variables mentioned in the section “Data collection” were finally determined. These variables can be easily acquired by instruments such as radiometers, anemometers, rain gauges, and barometers, making streamflow forecasting in ungauged catchments easily accessible in practice.

ED-DLSTM used the encoder to generate feature vectors for each catchment, which enabled the model to be somehow “aware” of differences between the hydrologic behaviors of different catchments. In the experiments, cosine similarity,^53^ a common measure in machine learning to assess feature similarity, is utilized to evaluate the coding ability of multiple models on the same catchments. It reflects how the models gauge the homogeneity and heterogeneity of different catchments and whether they can learn general hydrological patterns, as shown in Equation 15. Here, α and β represent the encoding vectors of the α and B catchments, respectively.

(Equation 15) $$\cos\left(\alpha ,\beta \right)=\frac{\alpha \cdot \beta }{{\left\|\alpha \right\|}_{2}{\left\|\beta \right\|}_{2}}$$

We selected 160 new catchments from the Chile dataset for a model transferability test to verify the model’s ability to generalize to ungauged catchments and evaluated internal parameter similarity through Equation 15. These catchments range in area from tens to thousands of square meters and belong to different HUC units, ensuring data diversity.

### Evaluation metrics

The performance metrics of the experimental results included the Pierce correlation coefficient (R), the Nash efficiency coefficient (NSE), the runoff ratio bias (RR), the root-mean-square error (RMSE), and the peak error ratio (FHV).^54^ The mathematical equations of the statistical indicators are described below. $Q_{o}^{t}$, $Q_{p}^{t}$, $\bar{Q_{o}}$, and $\bar{Q_{p}}$ are the observed, predicted, mean observed, and mean predicted values, respectively. $Q_{p}^{h}$ and $Q_{o}^{h}$ are the highest streamflow exceedance probabilities within the predicted and observed values, respectively. Here *h=1, 2 …, H* are the flow indices for flows with exceedance probabilities below 0.02.

(Equation 16) $$R=\frac{\sum_{t=1}^{T}\left(Q_{o}^{t}-\bar{Q_{o}}\right)\left(Q_{P}^{t}-\bar{Q_{P}}\right)}{\sqrt{\sum_{t=1}^{T}{\left({Q^{t}}_{o}-\bar{Q_{o}}\right)}^{2}}\sqrt{\sum_{t=1}^{T}{\left({Q^{t}}_{p}-\bar{Q_{p}}\right)}^{2}}}$$

(Equation 17) $$NSE=1-\frac{\sum_{t=1}^{T}{\left(Q_{o}^{t}-Q_{p}^{t}\right)}^{2}}{\sum_{t=1}^{T}{\left(Q_{o}^{t}-\bar{Q_{o}^{}}\right)}^{2}}$$

(Equation 18) $$RR=\frac{\sum_{t=1}^{T}\left(Q_{p}^{t}-Q_{o}^{t}\right)}{\sum_{t=1}^{T}Q_{o}^{t}}\times 100$$

(Equation 19) $$RMSE=\sqrt{\frac{1}{T}\sum_{t=1}^{T}{\left({Q^{t}}_{o}-{Q^{t}}_{p}\right)}^{2}}$$

(Equation 20) $$FHV=\frac{\sum_{h=1}^{H}\left(Q_{p}^{h}-Q_{o}^{h}\right)}{\sum_{h=1}^{H}Q_{o}^{h}}\times 100$$

## Data and code availability

ED-DLSTM uses both static and forcing data as inputs. All CSF tasks using ED-DLSTM should follow a similar dataset configuration template. Our data are sampled from the following:

Static data:

Digital elevation map (http://www.earthenv.org/DEM)
Snow cover extent (https://nsidc.org/data/myd10a1/versions/6)
Soil water content (https://cgiarcsi.community/data/global-high-resolution-soil-water-balance).
Groundwater table depth (http://science.sciencemag.org/content/339/6122/940)
Potential evapotranspiration (https://cgiarcsi.community/data/global-aridity-and-pet-database)
Aridity index (https://cgiarcsi.community/data/global-aridity-and-pet-database)

Forcing and streamflow observation data:

ERA5-Land reanalysis dataset (https://cds.climate.copernicus.eu/cdsapp#!/dataset/reanalysis-era5-single-levels?tab=overview)
The United States dataset (CAMELS) (https://ral.ucar.edu/solutions/products/camels),
Canadian Hydrometric dataset (HYSETS) (https://www.canada.ca/en/environment-climate-change/services/water-overview/quantity/monitoring/survey/data-products-services/national-archive-hydat.html).
The United Kingdom dataset (CAMELS-GB) (https://catalogue.ceh.ac.uk/documents/8344e4f3-d2ea-44f5-8afa-86d2987543a9)
Central Europe dataset (LamaH-CE) (https://zenodo.org/records/5153305)
Chile dataset (CAMELS-CL) (https://camels.cr2.cl/)

## Lead contact website

https://www.researchgate.net/profile/Chaojun-Ouyang.

## Supplemental information

Document S1. Figures S1 and S2 and Tables S1 and S2

Document S2. Article plus supplemental information
