# Supplementary material for: Deep learning for cross-region streamflow and flood forecasting at a global scale
Source: Innovation (Camb). 2024 Mar 26;5(3):100617. doi: 10.1016/j.xinn.2024.100617 (PMC11639694; doi:10.1016/j.xinn.2024.100617)
Supplement: Document S1. Figures S1 and S2 and Tables S1 and S2 [file mmc1.pdf]

The Innovation, Volume *5*

## Supplemental Information

# Deep learning for cross-region streamflow and flood forecasting at a global scale

**Binlan Zhang, Chaojun Ouyang, Peng Cui, Qingsong Xu, Dongpo Wang, Fei Zhang, Zhong Li, Linfeng Fan, Marco Lovati, Yanling Liu, and Qianqian Zhang**

# Supplemental Information

# Deep Learning for Cross-Region Streamflow and Flood Forecasting at a Global Scale

**Binlan Zhang, Chaojun Ouyang, Peng Cui, Qingsong Xu, Dongpo Wang, Fei Zhang, Zhong Li, Linfeng Fan, Marco Lovati, Yanling Liu, Qianqian Zhang**

## Table of Contents

**Figure S1.** The observation and prediction of streamflow from period t+1~t+5. Here t+1 and t+5 refer to the next day t+1 and the next five days t+5, respectively.

**Figure S2.** Differences in input modalities between ED-DLSTM and previous deep learning models.

**Table S1.** Results of ED-DLSTM and the benchmarks at different time steps.

**Table S2.** Dataset configuration for ED-DLSTM.

Figure S1 shows the model prediction of the hydrological station with ID 1333000 in CAMELS datasets as an example. The model prediction lead time includes the next one day to the next five day (t+1 to t+5). From time t+1 to t+5, the performance of the predictor gradually decreased, but the results are very excellent ($R>0.7$). For example, for the peak discharge in April 2011, the model can provide accurate predictions at time t+1 to t+3, underestimating and overestimating the streamflow at time t+4, t+5, respectively. This is reasonable because different penalty coefficients are used for different lead times, and this is a process of increasing entropy. It is worth noting that during the two-year testing process, the prediction model shows outstanding predictions for stable flow in most periods. But for the prediction of annual maximum flood, the predicted values are all higher than the observed values. In the period before the peak flow, the rainfall also reached the maximum value, indicating that the model is sensitive to the rainfall parameter in this catchment.

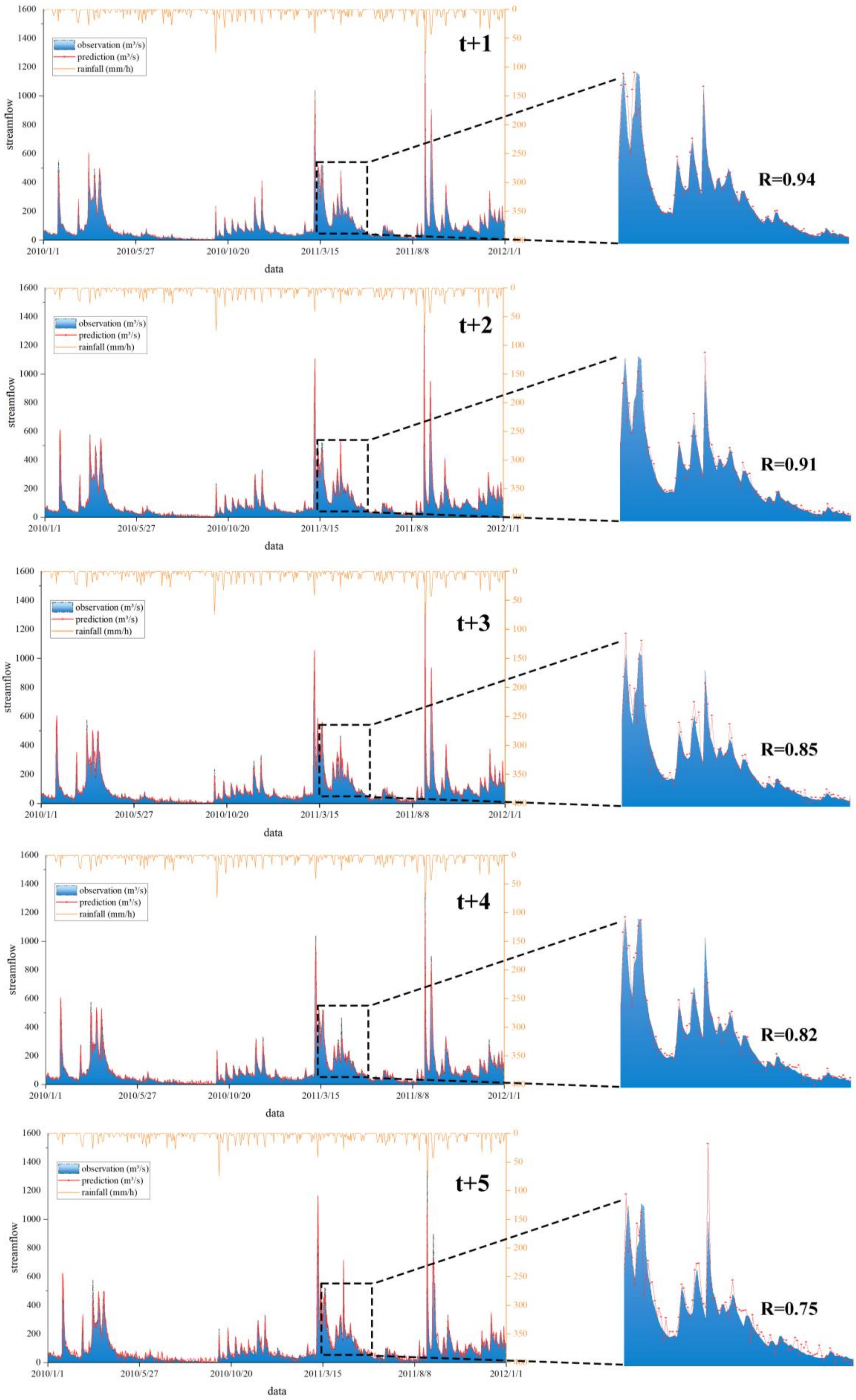
observation (m³/s)
prediction (m³/s)
rainfall (mm/h)
streamflow
data
2010/1/1
2010/5/27
2010/10/20
2011/3/15
2011/8/8
2012/1/1
t+1
R=0.94
t+2
R=0.91
t+3
R=0.85
t+4
R=0.82
t+5
R=0.75

**Figure S1.** The observation and prediction of streamflow from period t+1~t+5. Here t+1 and t+5 refer to the next day t+1 and the next five days t+5, respectively.

ED-DLSTM includes a module that encodes spatial raster properties, which allows it to learn static attributes spatially using raw remote sensing products instead of an aggregation index like Kratzert[18] did. Figure S2 takes soil moisture content (unit: %) as an example to show the difference in input.

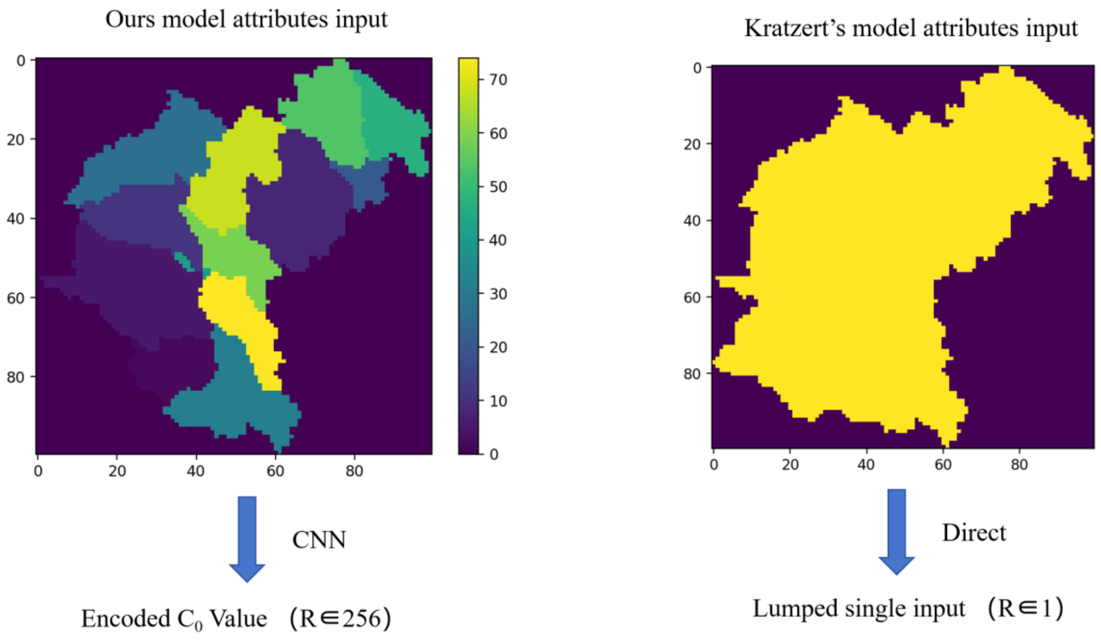


**Figure S2.** Differences in input modalities between ED-DLSTM and previous deep learning models.

The comparison of prediction results between ED-DLSTM and the benchmarks at different time steps is shown in Table S1. ED-DLSTM achieved the best performance in most cases. Among the indicators, larger values are preferred for indicators R, NSE, while a smaller value is preferred for indicator RR, RMSE, FHV.

**Table S1** Results of ED-DLSTM and the benchmarks at different time steps.

| | t+1 | | | | | t+2 | | | | | t+3 | | | | | t+4 | | | | | t+5 | | | | |
|---|---|---|---|---|---|---|---|---|---|---|---|---|---|---|---|---|---|---|---|---|---|---|---|---|---|
| Method | R | NSE | RR | RMSE | FHV | R | NSE | RR | RMSE | FHV | R | NSE | RR | RMSE | FHV | R | NSE | RR | RMSE | FHV | R | NSE | RR | RMSE | FHV |
| ED-DLSTM | **0.827** | **0.793** | **4.45** | **9.044** | 18.84 | **0.821** | **0.768** | 5.33 | **11.045** | 19.53 | **0.793** | **0.705** | **5.88** | **13.719** | 22.01 | **0.754** | 0.641 | **7.21** | **15.810** | 23.12 | **0.717** | **0.610** | **10.24** | **17.214** | **23.67** |
| CNN-LSTM | 0.784 | 0.701 | 4.97 | 12.738 | 17.51 | 0.766 | 0.693 | **5.21** | 13.562 | 19.01 | 0.759 | 0.680 | 6.03 | 15.394 | 22.43 | 0.741 | **0.674** | 8.27 | 17.691 | 24.88 | 0.695 | 0.595 | 10.98 | 19.206 | 24.12 |
| ARIMA | 0.715 | 0.548 | 11.34 | 15.956 | 18.93 | 0.701 | 0.501 | 11.90 | 17.593 | 23.11 | 0.688 | 0.427 | 12.78 | 20.640 | 25.46 | 0.642 | 0.409 | 13.56 | 23.507 | 30.46 | 0.604 | 0.388 | 16.80 | 25.311 | 34.77 |
| BLR | 0.639 | 0.423 | 17.63 | 24.690 | 21.15 | 0.620 | 0.402 | 19.21 | 26.334 | 26.80 | 0.604 | 0.343 | 23.15 | 31.745 | 27.41 | 0.587 | 0.210 | 28.90 | 36.829 | 31.24 | 0.542 | 0.196 | 31.03 | 40.918 | 35.69 |
| FCN | 0.754 | 0.650 | 8.68 | 13.009 | 15.67 | 0.717 | 0.607 | 8.90 | 15.082 | 17.55 | 0.703 | 0.588 | 10.05 | 17.884 | **21.83** | 0.694 | 0.521 | 11.26 | 21.101 | **23.00** | 0.689 | 0.425 | 14.69 | 24.006 | 25.14 |
| SAC-SMA | 0.760 | 0.646 | 8.79 | 14.287 | **11.98** | 0.704 | 0.584 | 10.03 | 16.369 | **16.97** | 0.682 | 0.546 | 11.84 | 19.210 | 23.30 | 0.663 | 0.422 | 15.59 | 22.934 | 25.86 | 0.594 | 0.407 | 18.37 | 24.927 | 26.50 |

We systematically divided the data into three parts: the training set, the testing set, and the validation set, as shown in table S2. The training set of each region trains one model separately, aims to enable the model to learn hydrological relationships and latent features between among different regions, while the testing set and validation set are used for model generalization testing.

**Table S2** Dataset configuration for ED-DLSTM.

| Type | Region | Number of Catchments | Time Span |
|---|---|---|---|
| Training set | The United States, Canada, Central Europe, The United Kingdom | 2089 | January 1, 1981 to December 31, 2009 |
| Testing set | The United States, Canada, Central Europe, The United Kingdom | 2089 | January 1, 2010 to January 1, 2012 |
| Validation set | Chile | 160 | January 1, 2010 to January 1, 2012 |
